# A Multi‐Method Exploration of the Support Needs of Registered Undergraduate Students of Nursing or Midwifery Transitioning Into the Workforce

**DOI:** 10.1002/nop2.70431

**Published:** 2026-03-09

**Authors:** Vanessa J. Watkins, Tanita Botha, Angela Erwin, Susan Perlen, Linda Sweet, Nicole M. Phillips, Debra Kerr, Alison M. Hutchinson

**Affiliations:** ^1^ School of Nursing and Midwifery Deakin University Geelong Australia; ^2^ Centre for Quality and Patient Safety Research Institute for Health Transformation, Deakin University Geelong Australia; ^3^ Biostatistics Unit Faculty of Health, Deakin University Geelong Australia; ^4^ Barwon Health Geelong Australia; ^5^ Western Health Melbourne Australia

**Keywords:** health services research, midwifery, nursing students, nursing workforce, workforce planning

## Abstract

**Background:**

The Registered Undergraduate Students of Nursing or Midwifery (RUSON/M) employment model was introduced in hospitals in Victoria, Australia, to provide supernumerary assistance to support patient care under the supervision of registered nurses and midwives. However, there is limited understanding of the support needs of students to transition into employment within the health workforce.

**Aim:**

To explore the support and resource needs of RUSON/M to facilitate transition into the health workforce.

**Design:**

Sequential multi‐method, exploratory, descriptive study.

**Methods:**

Surveys were conducted with RUSON/M, and semi‐structured interviews were undertaken with employed students and registered nurses and midwives within a public health service in Victoria, Australia. Data analyses included descriptive statistics (surveys) and inductive thematic analysis (interviews).

**Results:**

Forty Registered Undergraduate Students of Nursing (RUSON) and 10 Registered Undergraduate Students of Midwifery (RUSOM) participated in the survey (response rate 32.3%). RUSOM indicated stronger agreement with feeling supported than RUSON (median 2.0, IQR 1.0–2.0 and median 3.0, IQR 2.0–3.0, *p* = 0.0040, respectively). Similarly, RUSON participants expressed lower confidence (median 2.0, IQR 1.0–2.0) compared to RUSOM participants (median 1.0, IQR 1.0–1.0, *p* = 0.00859). Twelve RUSON/M and 22 registered nurses or midwives participated in an interview, in which it was identified that the undergraduate employment role is beneficial to the delivery of patient care. RUSON/M valued exposure to a wide range of clinical experiences to build their confidence in clinical skills and improve their work readiness upon graduation. Continuity in workplace allocation facilitated relationship‐building, supported skill competency assessment, built trust in capabilities and enabled timely debriefing.

**Conclusion:**

RUSON/M models confer benefits to patients, the workforce and the health service. The findings highlight the need for an approach to their employment that fosters the development of professional relationships, incorporates clinical leadership and mentorship and provides timely support to enable employed undergraduate students to have a positive experience.

**Implications for the Profession and/or Patient Care:**

Optimal undergraduate employment models provide benefits to patients, the workforce and the health service by relieving workload pressure.

**Impact:**

Mentorship enables undergraduate students to become effective members of the healthcare system workforce. To optimise benefits, models of undergraduate student deployment should enable the development of clinical leadership and professional relationships within teams.

**Reporting Method:**

Standards for Reporting Qualitative Research (SRQR) guidelines and Strengthening the Reporting of Observational Studies in Epidemiology (STROBE) guidelines.

**Patient or Public Contribution:**

None.

## 1 Introduction

Registered Undergraduate Students of Nursing (RUSON) and Registered Undergraduate Students of Midwifery (RUSOM) are employed in Victorian hospitals in Australia as a supplemental workforce under the supervision and delegation of registered nurses and midwives. All students enrolled in programs approved by the Nursing and Midwifery Board of Australia (NMBA) are registered as students with the NMBA. Nursing students in the second or third year of study in a program leading to registration as a registered nurse or midwife are eligible to apply for employment in a RUSON or RUSOM role.

Employment of undergraduate student nurses within a health service to assist registered nurses in delivering delegated aspects of patient care was initially trialled in 2016 (Victorian State Government Department of Health 2023). This trial evolved into the current RUSON model and was expanded during the COVID‐19 pandemic to include employment of undergraduate midwifery students in their final year as RUSOM to support patient and family care in the context of increasing acuity, faster pace and higher demand (Topple et al. 2023; Wynter et al. 2021).

Nurses and midwives are the largest group of registered health professionals in Australia (Australian Institute of Health and Welfare 2024). The relative decline in the nursing and midwifery workforce in Australia represents a major risk for health services, with predictions of significant workforce shortages in the future. Modelling conducted on the Australian nursing workforce indicates a projected undersupply of 70,707 nurses and the need for 79,473 new nurses in Australia by 2035 (Australian Government 2024b). For the midwifery workforce, the ratio of registered midwives per 100,000 head of population has dropped by almost 4% in the past five years, despite a growing population (Homer et al. 2024). The need to recruit and retain nurses and midwives in the health workforce remains critical (Australian Government 2024a). The Victorian State Government included the RUSON and RUSOM roles (RUSON/M hereafter when referring to both RUSON and RUSOM) in the workforce strategy to build stepped career paths while supporting health professionals to operate to the top of their scope (Victorian State Government Department of Health 2024b).

## 2 Background

Undergraduate nursing and midwifery students have been employed for several years and in diverse roles to supplement the healthcare workforce around the world. Several undergraduate nursing or midwifery student employment models exist within Australia that are either regulated or unregulated by the Australian Health Practitioner Regulation Agency (Ahpra) (Lindsay et al. 2023). In the state of Victoria, RUSON/M employment models are regulated by Ahpra (Victorian State Government Department of Health 2023), as is the Undergraduate Student in Nursing (USiN) program trialled in the state of Queensland (Lindsay et al. 2023; Raffelt 2018). Undergraduate nursing or midwifery students may also engage in paid employment through workforce models that are not currently regulated by Ahpra, such as Assistants in Nursing (AIN), Assistants in Midwifery (AIM) and Personal Care Worker (PCW) roles (Lindsay et al. 2023).

Employment of RUSON/M was endorsed and partly sponsored by the Victorian Government during the COVID‐19 pandemic as a flexible workforce to address predicted staffing shortages (Victorian State Government Department of Health 2023). The RUSON/M roles, embedded within Victorian public health services, continue to evolve to address the workforce needs of each individual health service (Victorian State Government Department of Health 2024a). There are currently over 50 RUSON/M programs across Victoria (Victorian State Government Department of Health 2024a).

Current empirical literature exploring undergraduate nursing or midwifery student employment models has largely focused on their demographic, education and health‐related characteristics (Warner et al. 2020), role scope and skill development (Algoso et al. 2018; Burns et al. 2022), employment intentions (Clynes et al. 2020) and feasibility and outcomes of the employment role (Kenny et al. 2021; Mumford et al. 2023; Willetts et al. 2021). Other studies have explored experiences of balancing study and work (Mitchell 2020), mental health experiences during the COVID‐19 pandemic (Wynter et al. 2021) and how their employment prepares them for future roles as registered nurses or midwives (Mumford et al. 2023; Sweet et al. 2023). Little is known about RUSON/M experiences, during their employment in the role, or of support to assimilate into the workforce and healthcare setting. The aim of this study was to explore the support, information and resource needs of RUSON/M to facilitate transition into the health workforce.

## 3 Methods/Methodology

### 3.1 Study Design

A sequential multi‐method exploratory, descriptive study design was used (Creswell and Plano Clark 2017). A cross‐sectional survey and individual semi‐structured interviews were conducted.

### 3.2 Study Setting, Population and Recruitment

#### 3.2.1 Setting

The study was conducted in a large health service in Victoria, Australia. The health service provides a comprehensive range of both acute and subacute services. Acute services include emergency and critical care, surgical, medical, oncology, maternity and paediatrics. Subacute services include inpatient rehabilitation, transitional aged care and mental health. The health service has collaborative partnerships with several universities and higher education providers that deliver undergraduate and postgraduate education for a range of health professionals.

#### 3.2.2 Population

The population for this study included all RUSON/M, as well as Registered Nurses and Midwives employed at the health service during 2022/2023 with experience of supervising RUSON or RUSOM in the workplace. The participant inclusion and exclusion criteria are detailed in Table 1.

**TABLE 1:** Participant inclusion and exclusion criteria.

Inclusion criteria	Exclusion criteria
RUSON and RUSOM employed by the health service Registered nurses and midwives employed by the health service with experience supervising or working with RUSON and/or RUSOM employed at the health service	RUSON or RUSOM not employed by the health service Enrolled nurses or unregistered care personnel providing nursing care employed by the health service Nurses and midwives employed by the health service with no experience of either supervising or working with RUSON and/or RUSOM employed at the health service Nurses and midwives not employed by the health service

#### 3.2.3 Recruitment

All RUSON (*n* = 136) and RUSOM (*n* = 19) employed at the health service were invited to participate in the study by completing a survey and/or participating in an interview to elicit their perceptions and experiences of systems and resources to support them in the workplace. Health service managers disseminated the invitation to all RUSON/M via their work email, including a plain language statement (PLS) with information about the study. At the end of the survey, RUSON/M participants were invited to contact the research team if they were also interested in participating in an interview. On behalf of the research team, managers disseminated a recruitment email to registered nurses and midwives employed in wards and units in which RUSON/M were employed. Included in the email were an electronic copy of the PLS and the contact details of the research team for individuals to register their interest in the study and self‐nominate to participate in an interview.

All RUSON/M, registered nurses and midwives who volunteered to participate in an interview were interviewed, and recruitment continued until information power (Malterud et al. 2016) was reached, which is the point at which no additional information is elicited. An AUD$30 gift card was provided to all interview participants to compensate for their time.

### 3.3 Data Collection

An electronic cross‐sectional survey was created in Qualtrics, a secure online platform. The survey incorporated the evaluation of the Rural Undergraduate Student of Nursing Employment tool (Kenny et al. 2021) that was used in the original evaluation of the RUSON pilot program. The survey requires respondents to rate their level of agreement with five statements using a 4‐point Likert scale ranging from Strongly Agree (1) to Strongly Disagree (4). These statements explored whether the RUSON and RUSOM scopes of practice were clear, the position description covered the expectations of the role, RUSON and RUSOM perceptions of support in their role and their confidence in completing activities delegated to them (Kenny et al. 2021).

Interview participants were offered the choice of participating either face‐to‐face or online via a video link. All interviews were conducted online and audio recorded. An interview guide with researcher‐developed questions was used to explore participants' perceptions, experiences and support needs of the RUSON/M role within the health service (Appendix A). Interview data were de‐identified, and audio files were transcribed verbatim by a professional transcription service. A member of the research team listened to the audio files to check the accuracy of the transcription, and participants were invited to check their transcript prior to de‐identification and analysis (Mero‐Jaffe 2011). No participants requested amendments to their transcripts.

### 3.4 Data Analysis

Descriptive statistics (frequency, percentage, mean and standard deviation) were utilised to summarise participant characteristics, with median and interquartile range (IQR) reported for questions relating to experience working within the health service. To assess the normality of the data, the Shapiro–Wilk test was conducted. As the data did not meet the assumption of normality, the Mann–Whitney *U* test was employed to compare the experiences of RUSON and RUSOM participants. To control for potential inflation of Type I error due to multiple testing, *p*‐values from the five planned significance tests were adjusted using the Bonferroni correction. For completeness, effect sizes (rank‐biserial correlations) were calculated for all Mann–Whitney *U* tests. Given the small sample size, these estimates should be interpreted with caution due to the increased uncertainty and potential instability of the values. All statistical analyses were performed using R software (version 4.2.2, R Foundation for Statistical Computing) (R Core Team 2024) with a significance level set at 5%.

Inductive coding and thematic analysis of the interview data were conducted in NVivo (Lumivero 2023), a qualitative data management software program. Thematic analysis followed Braun and Clarke's methods for reflexive thematic analysis (Braun and Clarke 2021, 2022, 2024). This analysis was conducted by three members of the research team (VW, SP and AH), and interpretations were reflected upon and discussed until consensus was reached. Initially, transcripts were read and re‐read to understand the meanings and to develop codes for segments of the data. Broad themes and subthemes were developed collaboratively by reflection upon the codes. These themes and subthemes were then presented back to a group of study participants for feedback on the authenticity of the findings, and this feedback was reviewed by the broader research team to interpret the findings in the context of the research objectives. To integrate and interpret the qualitative and quantitative findings, the survey and interview findings were compared to identify common and/or disparate findings, and instances where the qualitative findings could elaborate on or extend the quantitative results (Creswell and Plano Clark 2017).

### 3.5 Ethical Considerations

This research was conducted in accordance with the National Statement on Ethical Conduct in Human Research (National Health and Medical Research Council 2023). Ethical and governance approval was obtained from the human research ethics committee (HREC) of the health service and the HREC of Deakin University. All interview participants were given a copy of the participant information and consent form, and they provided either written or verbal consent at the beginning of the interview. All survey data were anonymous, and interview data were de‐identified and kept strictly confidential. Data were stored on a password‐protected secure server at the university. Interview participants were allocated an identifier code, used in the presentation of exemplar quotes in the findings section.

### 3.6 Rigour and Reflexivity

Strategies to enhance rigour and ensure the trustworthiness of the findings were employed throughout the research process. This was achieved by reflecting on factors that may introduce positivistic bias into the analytical process and interpretation of findings by the research team (Braun and Clarke 2023). To mitigate the potential risk of unequal relationships, the interviews were conducted by a research team member (VW) with extensive experience in conducting interviews for qualitative research and in supporting student and novice nurses and midwives in clinical practice. The interviewer was neither an employee of the health service nor involved in the delivery of the undergraduate nursing and/or midwifery courses.

## 4 Findings

### 4.1 Characteristics of Participants

A total of 50 RUSON/M responded to the survey (response rate 50/155, 32.3%), of whom 40 were employed as a RUSON (response rate 40/136, 29.4%), and 10 were employed as a RUSOM (response rate 10/19, 52.6%). Characteristics of the survey respondents are presented in Table 2. A total of 34 participants were interviewed, of which 12 (35.3%) were RUSON/M (8 RUSON and 4 RUSOM) and 22 (64.7%) were registered nurses or midwives. The nurses and midwives interviewed represented a range of clinical specialisms, levels of experience, and included some employed in leadership roles.

**TABLE 2:** Survey participant characteristics.

	All	RUSOM	RUSON
	(*n* = 50)	(*n* = 10)	(*n* = 40)
Gender^a^			
Female	35 (94.6%)	9 (100.0%)	26 (92.9%)
Male	2 (5.4%)	0 (0.0%)	2 (7.1%)
Age^a^			
Mean (SD)	26.05 (±7.43)	22.89 (±1.17)	27.07 (±8.29)
Region of birth^a^			
Australia	33 (89.2%)	9 (100.0%)	24 (85.7%)
North Africa, Middle East or SE Asia	3 (8.1%)	0 (0.0%)	3 (10.7%)
Prefer not to say	1 (2.7%)	0 (0.0%)	1 (3.6%)
Language spoken at home^a^			
English	32 (88.9%)	9 (100.0%)	23 (85.2%)
Dinka, Arabic, Gujarati, Portuguese, Punjabi and mixed English	4 (11.1%)	0 (0.0%)	4 (14.8%)
First Nations identification^a^			
Yes	0 (0.0%)	0 (0.0%)	0 (0.0%)
No	37 (100.0%)	9 (100.0%)	28 (100.0%)

^a^ Missing values (RUSOM = 1 and RUSON *n* = 12).

### 4.2 Survey Results

The categorical responses to the five survey questions are presented in Table 3. Statistically significant differences were identified in RUSON and RUSOM participant responses to two questions. In response to the statement *‘I felt well supported in my role as RUSON/M’*, RUSOM participants (median 2.0, IQR 1.0–2.0) reported higher provision of support compared to RUSON participants (median 3.0, IQR 2.0–3.0, *p* = 0.0040, adjusted *p*=0.0198 rank‐biserial effect size = 0.56) indicating a medium‐to‐large difference. Similarly, RUSOM participants expressed higher confidence (median 1.0, IQR) compared to RUSON participants (median 1.0, IQR 1.0–1.0, *p* = 0.0085, adjusted *p* = 0.0424, rank‐biserial effect size = 0.48) in response to the statement ‘*I felt confident in completing the activities delegated to me as RUSON/M’, reflecting medium effect*.

**TABLE 3:** RUSOM experiences of working within the health service^a^ compared to RUSON.

Question	RUSOM			RUSON			*p*	Adjusted *p*	Effect size
	Min	Max	Median (IQR)	Min	Max	Median (IQR)			
The scope of practice of the RUSON/M was clear	1	3	2.0 (2.0–2.0)	1	4	2.0 (1.8–2.0)	0.9999	0.9999	0.00
The RUSON/M job position description covered the expectations of the role	1	4	2.0 (1.0–2.8)	1	4	2.0 (1.0–2.0)	0.8753	0.9999	0.03
I felt well supported in my role as RUSON/M	1	3	2.0 (1.0–2.0)	1	4	3.0 (2.0–3.0)	0.0040**	0.0198*	0.56
I felt confident in completing the activities delegated to me as RUSON/M	1	2	1.0 (1.0–1.0)	1	3	2.0 (1.0–2.0)	0.0085**	0.0424**	0.48
The RUSON/M role has increased my intention for regional practice	1	2	1.0 (1.0–2.0)	1	4	2.0 (1.0–3.0)	0.0999	0.4997	0.32

^a^ Items taken from the Evaluation of the Rural Undergraduate Student Employment (RUSON pilot program) tool (Kenny et al. 2021).

*p‐value of ≤ 0.05 is statistically significant. **p‐value of ≤ 0.01 is highly statistically significant.

### 4.3 Interview Findings

**FIGURE 1:**
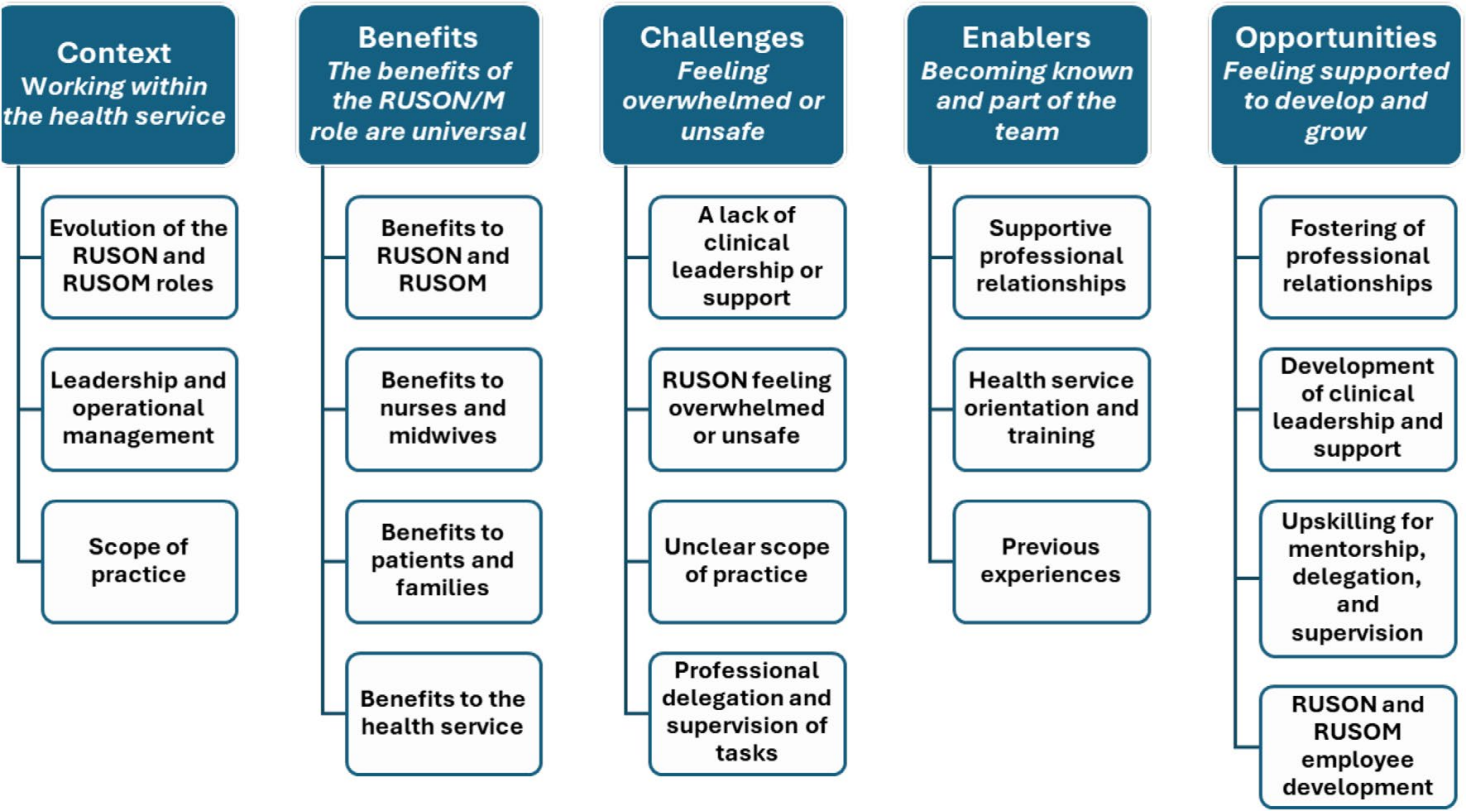
Major themes and subthemes of the interview findings.

Five major themes were identified following inductive thematic analysis: *Context—Working within the health service, Benefits—The benefits of the RUSON and RUSOM roles are universal, Challenges—Feeling overwhelmed or unsafe, Enablers—Becoming known and part of the team* and *Opportunities—Feeling supported to develop and grow*. The five key themes and subthemes are shown in Figure 1. Illustrative quotes for each theme and subtheme are presented in Table 4.

**TABLE 4:** Major themes and subthemes of the interview findings with illustrative quotes.

Major theme	Subtheme	Illustrative quotes
Context—Working within the health service	Evolution of the RUSON and RUSOM roles	‘It started out you're not allowed to be a CPO [Constant Patient Observer]. You're not allowed to cover breaks, things like that.… Then slowly they said, ‘okay, you can cover breaks, but you can't do a whole shift’. Then these days … I had five shifts in a row where … ‘okay, you're the CPO for this patient for the shift’ … We're paid less than a CPO … You feel like they're cheating … they're putting in the student who doesn't necessarily have the scope to do it, but they get it cheaper. It just feels a little bit wrong.’ (Participant 1, RUSON)
	Leadership and operational management	‘So, the first person I am reporting to is the midwife in charge on the ward that day. … I know I have to tell them what's happened, I get most of my support from the midwifery unit and team themselves than I do … the people who actually manage us.’ (Participant 13, RUSOM)
		‘I just sat in a room by myself and cried for 10 or 15 min. I just tried to gather myself … I wanted to be professional … But it was just the fact that I was new, and I didn't know what I was doing. I didn't know who to contact or what support to get. I didn't know how to do a RiskMan (A software tool on which risk‐related incidents are reported.), things like that … it isn't management's fault because they honestly didn't know.’ (Participant 10, RUSON)
	Scope of practice	‘So that's like a secret weapon … they get these more organic experiences where … they can develop their interdisciplinary collegialism … those incidental conversations that occur are the ones that make them feel part of the team or that kind of gets them to see how the team works and interacts. I'd hate for them just to be used for re‐stocking … I don't know whether that's a really good way for them to get experiences.’ (Participant 15, RN)
Benefits—The benefits of the RUSON and RUSOM role are universal	Benefits to RUSON and RUSOM	‘With breastfeeding … I feel like the actual RUSOM job itself helped 10 times more than the theory side because what you can apply to one person might be different to someone else. I think the experience that I've got as a RUSOM has definitely helped me a lot more … being a lot more confident and helping women breastfeed than I learnt at uni.’ (Participant 11, RUSOM)
		‘I've been able to see a little bit of cardiology, a bit of surgical, a bit of medical, rehabilitation, really pretty much a mix of everything, which has been really helpful for me knowing for my future maybe where I'd like to prioritise my work’ (Participant 2, RUSON)
	Benefits to registered nurses and midwives	‘In terms of [the midwives'] mental health … staff feel like their workload is easier when there's a RUSOM around to support the acuity, and I know that midwives also are able to get breaks easier if there is a RUSOM around … It allows the midwife to either sit down and write notes, have a break or get off their shift on time … We might not have done everything that we wanted to, but we've done enough to be able to go home and switch off.’ (Participant 27, RM)
		‘It's like an extra pair of hands, definitely. It definitely reduces our workload.’ (Participant 21, RN)
	Benefits to patients and families	‘Just simple things like ordering an air mattress in a timely manner … Being able to have the time to sit with the patient and ask them if they've got any questions post the doctor's review.’ (Participant 18, RN)
		‘Having RUSOMs around who could do so many of those simpler tasks … was amazing. It freed up the midwives to be able to do the midwifery stuff that they really needed to get done … Most of them [RUSOM] can sit down and go through our whole education sheet with the mums … [they] can sit and spend 45 min with a woman explaining things.’ (Participant 19, RM)
	Benefits to the health service	‘We as an organisation definitely benefit from their experience and their confidence … I believe our workforce would suffer without them.’ (Participant 28, RM)
		‘I've taken the idea of I'm here to help the nurses. So, if I'm like—oh today, I was like, hey, I see that someone's going to be discharged at 10:00, do you want me to shower her and get her ready to go home?’ (Participant 3, RUSON)
Challenges—Feeling overwhelmed or unsafe	A lack of clinical leadership or support	‘The only time I see [the casual workforce management team] is if I'm asking for a shift change, which might be once every month or so … [They] don't know who I am, that sort of thing. It'd be nice if we had a bit more close contact with our managers. It'd make them a lot more approachable for when we have adverse situations. Things like that.’ (Participant 1, RUSON)
		‘[It] was very scary, as a junior, to have to report to the ANUM [associate nurse unit manager] who is the … busiest person on the ward … I think you probably spent more time with the PSAs [patient service assistant??]and the cleaners, because they were the ones also floating. If you had questions, you would go to them. Because they had the time to talk to you, normally. They can be a bit more approachable.’ (Participant 34, RUSON and RUSOM)
	RUSON feeling overwhelmed or unsafe	‘I know there's been at least two patients where I've had to let them roam the ward because they were getting angry if we tried to direct them to their bed or to their room. I've been in situations where they might try and swipe at you. Or I have ‐ recently in the past couple months, I got chased down a hallway and things like that. That's happened twice with different patients. It's just a little bit tricky. You feel quite a lot out of your depth.’ (Participant 1, RUSON)
		‘If a patient needs a CPO [constant patient observer] and … there is no other staff available, sometimes we have to call the RUSON … Once on my shift, I noticed that she [the RUSON] had never, ever done a CPO shift … it was actually challenging for her. I had to support her a lot. Because the patient had escalating behaviours … We had to call [for security support]. It was entirely new to her. She thought it was because of her mistake that we had to call [for security].’ (Participant 21, RN)
	Unclear scope of practice	I think they did end up changing our contract slightly to say that we could be a constant patient observer, because at the start, we didn't do that at all. That's when it started to get a little bit more grey about what patients we could take … it was very confusing. I'm still confused about what patients I can take. But yeah, at the start, it was very clearcut what you can and can't do, and now I would say it's a little more difficult to determine. (Participant 10, RUSON)
		‘To start off with it was a little bit—I suppose it was very—there was very much a grey area, about what they could and they couldn't do. I think that probably meant that—particularly in our ward, we often err on the side of caution [laughs]—so I think they probably came to our ward, and didn't get to do as much as what they're probably capable of doing’. (Participant 26, RN)
	Professional delegation and supervision of tasks	‘I know, initially, I was hesitant to delegate tasks to the RUSOM because it would come back to me if anything went pear‐shaped, I suppose … If you haven't worked with someone before, you don't necessarily have that trust there that they will work the same level as what you would want.’ (Participant 27, RM)
		‘You want to make sure that you're taking care of your patients and you're not missing anything and especially handing that over to someone more junior than yourself … I was quite mindful of doing that. I didn't want to let them do it, something be wrong, they not tell me and then it's my fault because then there's something wrong. So yeah, very tricky.’ (Participant 29, RM)
Enablers – Becoming known and part of the team	Supportive professional relationships	‘Learning who that person was, learning their capabilities and, you know, working with that. Because we don't all work the same.’ (Participant 27, RM)
		‘The relationship you build with the team you're working with, and the more they get to know you, and they know how you work, … your scope … increases a little bit … I know how to do that. They know that if anything's wrong, I would escalate that to them. So, they're willing to let me do that.’ (Participant 13, RUSOM)
		‘If I'm on a shift and if I don't know the RUSON or if I think they are new, I will take the time to have a chat with them. Just to see what their understanding is and what they are supposed to do … try to build that rapport. As for the next time, you know I have met this person … I know exactly where they are up to.’ (Participant 21, RN)
	Health service orientation and training	‘I think I was pretty excited to get an understanding of what the role entailed, and being in a hospital setting, and working in a hospital is very, very different. Going back, the onboarding was good.’ (Participant 22, RUSON)
		‘They went through a heap of different things like what you can do as a RUSOM, what you can't do as a RUSOM, … and then they discussed things like uniforms, pay details.’ (Participant 11, RUSOM)
		‘The job list was just there. It was literally written. Do the skips, do the baby baths. Stock this up. Fill these drawers … You just go in and follow the list.’ (Participant 34, RUSOM)
		‘There's no operations manual … It's being able to talk to people. I think if you don't have the emotional intelligence … it could be a bit daunting.’ (Participant 22, RUSON)
	Previous experiences	‘I'd had two placements before I got into second year… [That] was … really helpful, … learning how the hospital works, … and getting used to shift work. … I think the units that [the university] runs in first year help prepare … those really foundational skills you'd be using later on.’ (Participant 2, RUSON)
Opportunities—Feeling supported to develop and grow	Fostering of professional relationships	‘Having allocated RUSON for wards would be fantastic because then they would get to know the team and become integrated… you could set the expectations of your department… [RUSON] would integrate and become one of the team.’ (Participant 17, RN)
		‘A lot of our RUSOMs from last year have now commenced back as graduate midwives … It's been nice to see the same faces … You know the things that they know, and you know the things that they might not have had a chance to consolidate.’ (Participant 30, RM)
	Development of clinical leadership and support	‘RUSON don't have a home so who follows them up? … Yeah, it worries me that they don't have a—kind of a central educator or a central person … They haven't even started their career yet. We don't want them to have career‐ending experiences before they even start.’ (Participant 15, RN)
		‘We just want to look after them, and do they have enough support? I don't know if they do. Who actually looks after them? … There's not a clear pathway like there is with the students and the educators, because they're employees … not students.’ (Participant 31, RM)
	Upskilling for mentorship, delegation, and supervision	‘You have to work together to get your tasks done, but you are still directly allocated to four patients … that real delegation supervision is something that we are probably not great at … I think we get so stuck in that one‐to‐four mentality … So, while you can do it together with a RUSON, it's really just about the identification of what tasks they can do to minimise your workload and if you could find the task and then learn that it would reduce your workload by giving them away. … But that takes a little bit of effort and time, I suppose, where certain nurses are just so focused on the things they've got to do, and they just do it themselves.’ (Participant 16, RN)
		‘People weren't even really sure what their [RUSON] scope of practice was … I think if you made staff aware of what the RUSONs were capable of and what they could do, that people would probably delegate more often.’ (Participant 17, RN)
	RUSON and RUSOM employee development	‘Maybe a little more practice with things like a respiratory assessment or … with things like neurovascular obs, and wound charts, and things like that we don't really go over too much at uni, but it is helpful … I don't think I ever did a fluid balance chart in uni, and I do them all the time as a RUSON.’ (Participant 10, RUSON)
		‘I just think there's a real opportunity to showcase the skills of [the health service] …it has a really good reputation for grads …. Everyone is really happy there, the opportunity that they've got. But I think there's a big opportunity for them to grab hold of the current cohort of RUSONs to say, stay with us … where do you want to go? Like for me, personally, I would love to be able to sit down with someone ‐ not in the grad [graduate] team, like I've still got a couple of years left, but to go, this is what I want to do. How do I get there?’ (Participant 22, RUSON)

#### 4.3.1 Theme 1: Context—Working Within the Health Service

The *Context—Working within the health service* theme comprises three subthemes, reflecting the *Evolution of the RUSON and RUSOM roles* at the health service, the *Leadership and operational management* of the RUSON/M workforce and the *Scope of practice* of the RUSON/M workforce.

##### 4.3.1.1 Subtheme 1.1: Evolution of the RUSON and RUSOM Roles

Participants in leadership roles noted that the RUSON role was introduced in the health service prior to the COVID‐19 pandemic. Most discussed the rapid expansion of the RUSON role as part of the surge workforce to support the pandemic emergency response, followed by the introduction of the RUSOM role. Nurses, midwives and RUSON/M participants acknowledged that the orientation of RUSON/M to the health service during the COVID‐19 pandemic was challenged by competing demands and unpredictable surges in workload. Contributing factors included mandated restrictions on movement and gatherings to minimise transmission of COVID‐19 (State Government of Victoria 2022), which meant that standard workplace orientation programs held at the commencement of employment were cancelled. Extra education resources within the health service, funded by the Victorian Government's pandemic response to support the surge workforce, were highly valued by RUSON. Several RUSON expressed disappointment that these resources ended when the emergency pandemic response funding was withdrawn.

The RUSON role was reported to have evolved in the post‐pandemic period with the assignment of new tasks such as the Constant Patient Observer (CPO) role, in which a member of staff provides constant observation of one patient who has been assessed as at risk of harming themselves or others. Some RUSON expressed frustration with perceived inconsistency in remuneration following this change, as unregistered Health Care Workers (HCW) who previously fulfilled the CPO role received a higher wage than RUSON. In contrast, RUSOM participants did not identify changes in their employment role or duties over time.

##### 4.3.1.2 Subtheme 1.2: Leadership and Operational Management

Within the subtheme *of Leadership and operational management*, a system of centralised management enabled flexible deployment of RUSON to areas with high patient acuity or clinical need. Exposure to a wide range of clinical experiences in several wards was described as a benefit of this deployment model for RUSON. In contrast, participants reported that RUSOM were almost exclusively deployed to the post‐natal area and were rarely deployed to any other clinical environments.

Most registered nurses and midwives were unsure about the reporting line for RUSON/M. Timely access to line‐manager support for RUSON/M was recognised as a priority by all participants. It was reported that the administrative management team was less likely to be aware of potentially upsetting incidents or events in which a RUSON/M may have been involved. Several RUSON reported difficulty accessing support after experiencing distressing events.

##### 4.3.1.3 Subtheme 1.3: Scope of Practice

Within the subtheme *Scope of practice*, participants explained that the initial RUSON and RUSOM scope of practice had been negotiated between the Victorian Department of Health, the nursing and midwifery trade union (Australian Nursing and Midwifery Federation), and public health services. Participants agreed that maintaining professional behaviour was important, and expectations for the scope of practice should be communicated across the organisation to ensure a consistent approach and high standard of care.

Registered nurses and midwives described themselves as professionally accountable and responsible for the delegation of clinical tasks and supervision of RUSON/M on shift. Most registered nurses and midwives described feeling a high level of confidence in their professional delegation and supervision role. However, many registered nurses and midwives also explained they were unclear about the expectations for RUSON and RUSOM scope of practice in different clinical contexts. Nevertheless, there was acknowledgement that RUSON and RUSOM appeared to understand and practice within their scope of practice. Several registered nurses expressed concern that RUSON were not always delegated tasks across the expanse of their scope of practice (i.e., they tended to be delegated more basic tasks).

#### 4.3.2 Theme 2: Benefits—The Benefits of the RUSON and RUSOM Role Are Universal

The theme *Benefits—The benefits of the RUSON and RUSOM role are universal* includes four subthemes: *Benefits to RUSON and RUSOM, Benefits to registered nurses and midwives, Benefits to patients and families*, and *Benefits to the health service*.

##### 4.3.2.1 Subtheme 2.1: Benefits to RUSON and RUSOM

Perceived *Benefits to RUSON and RUSOM* included improved clinical and interpersonal skills, flexible working hours and improved work readiness at graduation. RUSON and RUSOM valued the RUSON/M employment role because earning a wage was vital, which they had to balance with the competing demands of compulsory placement. All RUSOM identified continued exposure to the clinical environment with a group of trusted midwifery colleagues as beneficial to improving their confidence and feelings of work readiness. In contrast, RUSON reported deployment to a different ward each shift. Whilst most RUSON valued the exposure to varied experiences, many also described experiencing challenges, which are explored further under Theme 3.

##### 4.3.2.2 Subtheme 2.2: Benefits to Registered Nurses and Midwives

The RUSON/M were universally acknowledged as an asset to the working environment. Perceived *benefits to registered nurses and midwives* also included increased ability to manage workload and take breaks. Reliance on the RUSOM workforce to manage the workload and the improved sense of personal well‐being and workplace morale conferred by the work of RUSOM were emphasised by all midwife participants. All midwife participants also reported increased job satisfaction associated with confidence that the women and families were receiving the attention and education that they needed. Nurses and midwives also identified benefits to their professions from having future graduates with RUSON and RUSOM experience. It was anticipated by some that graduates would value the opportunity to interact with patients and women during their employment as a RUSON/M.

##### 4.3.2.3 Subtheme 2.3: Benefits to Patients and Families

*Benefits to patients and families* were reported by all groups of participants to include improved experience of care, timely assistance with personal care and comfort needs, provision of direct patient care and/or education, improved safety through timely clinical observation and escalation of care, and improved communication and psychological/emotional support. Nurses and midwives were clear in their views that the RUSON/M roles were useful to enhance care and should not be used to replace registered nurses or midwives during times of staff shortages. RUSON/M were particularly valued as a supplementary workforce to support vital, but time‐consuming, aspects of care such as patient education or support with infant care during transition to parenthood.

##### 4.3.2.4 Subtheme 2.4 Benefits to the Health Service

*Benefits to the health service* identified by all groups of participants included a flexible workforce that could be promptly deployed to meet clinical needs, reduced nursing and midwifery workforce stress and burnout, supporting retention and recruitment of nursing and midwifery staff, enhanced recruitment of future nurses and midwives, improved quality, safety and patient experience of care, and improved patient flow. Participants described the RUSON/M models as enabling a flexible workforce that allowed the organisation to adapt to changing circumstances and peaks in demand. This flexibility was highly valued during the peak of the pandemic. When extra beds were opened, the RUSON workforce provided hands‐on support to nurses in terms of the provision of basic patient care and sourcing of equipment. This was described as mutually beneficial through safely managing the workload, gaining unique experiences and feeling a valued member of the workforce. Midwife and RUSOM participants described the RUSOM as benefiting the health service through their support in the provision of maternity care to a growing population with higher acuity and increasing demand for services within the region.

#### 4.3.3 Theme 3: Challenges—Feeling Overwhelmed or Unsafe

Four subthemes are included in the theme, *Challenges—Feeling overwhelmed or unsafe*. The subthemes are *a lack of clinical leadership or support, RUSON feeling overwhelmed or unsafe, unclear scope of practice* and *professional delegation and supervision of tasks*.

##### 4.3.3.1 Subtheme 3.1: A Lack of Clinical Leadership or Support

The RUSON/M workforce was employed and managed through a centralised workforce management model for casual nurses, midwives and healthcare workers. Most participants suggested that it would be beneficial to develop a closer working relationship with the workforce managers. Challenges associated with a lack of clinical leadership or support were more pronounced in the experiences of RUSONs and nurses, as RUSONs were regularly deployed to different places. As a result, RUSONs were less likely to develop professional relationships with nurses on the wards. These experiences were not reported by RUSOMs or midwives.

##### 4.3.3.2 Subtheme 3.2: RUSON Feeling Overwhelmed or Unsafe

Most RUSON participants expressed feelings of isolation when working on some wards and the challenges associated with integration into the nursing team on a different ward each day. Several of the RUSON cohort explained that they sometimes felt overwhelmed or unsafe during their shift, and some also revealed a personal experience of physical assault while working in the RUSON role. Expression of feelings of vulnerability and being inadequately prepared for occupational violence and aggression was common amongst RUSON participants. Several registered nurses also expressed their concerns that RUSONs were under‐prepared to work in a CPO role, even though they were being deployed into these roles more frequently.

##### 4.3.3.3 Subtheme 3.3: Unclear Scope of Practice

Almost all participants described challenges associated with a perceived lack of clarity regarding the RUSON scope of practice in the different clinical contexts of the hospital and changes to the RUSON role within the hospital. Some RUSON participants expressed confusion about the tasks that were required of them in different clinical settings and feelings of being unhelpful as a result. Many of the nurse and RUSON participants perceived that the RUSON scope of practice became less clear when there was a shift in RUSON deployment in the hospital, with more RUSON being allocated to CPO or HCW roles.

##### 4.3.3.4 Subtheme 3.4: Professional Delegation and Supervision of Tasks

Many of the nurse and midwife participants expressed the opinion that delegation to individual RUSON and RUSOM was a learning curve and was different to their experiences of preceptorship of students on clinical placement. Several nurses and midwives described feeling cautious about the delegation of more complex tasks to RUSON and RUSOM with whom they had not worked before.

#### 4.3.4 Theme 4: Enablers—Becoming Known and Part of the Team

Three broad subthemes were identified under the theme, *Enablers—Becoming known and part of the team*. These subthemes are *Supportive professional relationships, Health service orientation and training*, and *Previous experiences*.

##### 4.3.4.1 Subtheme 4.1: Supportive Professional Relationships

The opportunity to develop professional relationships between individual nurses and midwives and RUSON/M colleagues was valued by all participants. RUSOMs reported that the development of relationships with midwifery colleagues facilitated the professional development of RUSOMs to gain valuable clinical experience and improved their confidence to provide effective breastfeeding support for women in their care.

##### 4.3.4.2 Subtheme 4.2: Health Service Orientation and Training

Although most RUSON/M who commenced work during the COVID‐19 pandemic described a poor experience of onboarding and orientation to the hospital, RUSON/M who were employed later expressed more favourable opinions. A number of structures that assisted in developing familiarity with the hospital and workplace were identified as enablers for RUSON/M in their transition into the workforce. Having a tour of the hospital during orientation was described as a positive experience, as was the opportunity to discuss workplace expectations with management staff. Having had a previous student placement at the hospital was also described by RUSON/M as helpful for orientation to their new RUSON/M employment roles. Similarly, RUSON/M reported that having a list of tasks they could complete each shift enabled them to assimilate into the workplace and feel useful to the nurses and midwives on shift.

##### 4.3.4.3 Subtheme 4.3: Previous Experiences

Attributes such as previous life experience and emotional maturity were considered by all participant groups as assets to enable RUSON/M to assimilate into their role and integrate into the nursing and midwifery teams. A RUSON described the importance of emotional intelligence to understand and evaluate what is required of the RUSON on each shift. Participants from all groups expressed the view that the knowledge and skills developed during undergraduate study were enablers of RUSON/M transition to practice. RUSON and RUSOM reported that the theoretical learning achieved at university and professional practice experience acquired during clinical placements in the hospital helped smooth the transition into the health service workforce and integration into their RUSON or RUSOM role. Suggestions were made for key skills, such as de‐escalation techniques, to be taught within the first year of the nursing and midwifery curriculum to prepare RUSON/M for the reality of clinical work.

#### 4.3.5 Theme 5: Opportunities—Feeling Supported to Develop and Grow

Within the *Opportunities—Feeling supported to develop and grow* theme, four subthemes were identified: *Fostering of professional relationships, Development of clinical leadership and support, Upskilling for mentorship, delegation and supervision*, and *RUSON and RUSOM employee development*.

##### 4.3.5.1 Subtheme 5.1: Fostering of Professional Relationships

Development of a RUSON workforce model that provides the opportunity for ward or department teams to get to know individual RUSON was recommended by most nurses. Having a system in which RUSON were allocated to regular areas was recommended as a strategy to help the RUSON feel part of the team, build relationships, and feel safer asking for support and guidance. Midwives and RUSOM participants described mutual benefits arising from the development of professional relationships between RUSOM and midwifery colleagues. These benefits included maximising opportunities for the advancement of midwifery skills for the RUSOM and for the development of confidence in the skills of individual RUSOM when delegating tasks to manage the midwifery workload.

##### 4.3.5.2 Subtheme 5.2: Development of Clinical Leadership and Support

Consideration of options to enable timely clinical support and debriefing within the health service for RUSON/M was recommended by all participants to bridge an identified gap in workforce support. Feelings of isolation were particularly voiced by the RUSON cohort, and a clear pathway for RUSON and RUSOM to access timely managerial or clinical leadership support at the point‐of‐care to meet education and/or debriefing needs was recommended as a priority.

##### 4.3.5.3 Subtheme 5.3: Upskilling for Mentorship, Delegation and Supervision

Several nurse and midwife participants reported their perceptions of a potential skills gap or lack of confidence relating to mentorship, delegation and supervision when working with RUSON/M. This was compounded by the preference of some nurses or midwives to independently manage their daily patient allocation. Some more junior nursing or midwifery participants disclosed feeling fearful of delegating tasks to RUSON or RUSOM as they were still gaining experience with managing their own workload. Nurses and midwives who identified as being employed in senior roles recognised variation in skill level for mentorship, delegation and supervision of RUSON/M; some of whom had already trialled some solutions such as a RUSON or RUSOM ward orientation package, in which tasks within RUSON or RUSOM scope of practice relevant to the speciality of the ward had been agreed and supports identified. However, one nurse further explained that the absence of staff training, particularly clarification of the RUSON scope of practice, continued to be a problem.

##### 4.3.5.4 Subtheme 5.4: RUSON and RUSOM Employee Development

To support RUSON/M and registered nursing and midwifery staff, the development of a credentialing system for core skills within the RUSON or RUSOM scope of practice was recommended. Proposed benefits of credentialling RUSON/M included the capacity for registered nurses and midwives to confidently allocate more complex tasks as RUSON and RUSOM developed their practice and consolidated their clinical skills.

## 5 Discussion

This study provided a unique insight into the support needs of RUSON/M regarding transition to their roles as undergraduate employees of a health service. Previous studies have explored the impact of undergraduate student paid employment models upon critical workforce shortages in midwifery (McLachlan et al. 2011), rural health (Kenny et al. 2021), mental health (Browne et al. 2013) and during the COVID‐19 pandemic emergency (Topple et al. 2023). Our findings provide further evidence of the range of benefits that RUSON/M employment models can offer undergraduate students, nurses and midwives, patients and families, and the health service. These benefits are consistent and build upon the findings of research conducted in other Victorian health services (Sweet et al. 2023; Victorian State Government Department of Health 2024a). This study confirms that the RUSON/M model is an innovative strategy to relieve workload pressures whilst strengthening the future nursing and midwifery workforce by improving professional confidence and work readiness upon graduation.

Other researchers have reported nurse satisfaction with the undergraduate employment role in terms of perceived improvements in workplace safety and patient satisfaction in paediatric care (Raffelt 2018), and for addressing non‐urgent care needs and early identification of clinical deterioration of patients (Willetts et al. 2021). Similarly, midwives have reported that employment of undergraduate midwifery students resulted in improvements in workload, enhancing the quality of care and outcomes for women and babies (Mumford et al. 2023) and giving midwives more time to work at the higher end of their scope of midwifery practice (Sweet et al. 2023). Previous research has focused on either the perceptions and experiences of undergraduate nurses employed in Assistant in Nursing (AIN) (Algoso and Peters 2012; Algoso et al. 2018, 2019) or RUSON roles (Kenny et al. 2021; Willetts et al. 2021) or the perceptions and experiences of undergraduate midwifery students employed in Assistant in Midwifery (AIM) (Burns et al. 2022) or RUSOM roles (Mumford et al. 2023; Sweet et al. 2023).

The unique contribution of this study lies in the inclusion of two different disciplines (nursing and midwifery), as there has been little research that has explored perspectives from both nursing and midwifery to date. In this study, key differences were identified in the experiences of nurses compared with midwives, which could be attributed to differences in the deployment strategies for RUSON compared to RUSOM. Survey findings in our study indicated that RUSON felt less supported than their RUSOM colleagues and less confident in performing tasks that were delegated to them. Through exploration of these findings in the interviews, we found that RUSOM experienced relative stability in their deployment in post‐natal wards, strongly contrasting with the experiences of RUSON being deployed to different wards on a regular basis. Continuity of RUSOM deployment to the post‐natal ward enabled the development of professional relationships between individual RUSOM and midwives. Professional relationships were recognised as mutually beneficial, as RUSOM described enhanced experiences of support and the development of professional confidence, and midwives reported the development of increased confidence in delegating more complex tasks within the RUSOM scope of practice to a known RUSOM. The benefits of continuity in workplace allocation identified in our research align with findings reported in a previous study of RUSOM (Sweet et al. 2023).

In contrast, RUSON participants deployed to different wards each shift often reported feeling unknown and/or ignored by their nursing colleagues. This was also reflected in the views of nurses who expressed low confidence in delegating complex tasks to RUSON who were unknown to them. Many RUSON reported feeling overwhelmed or unsafe, particularly after exposure to occupational violence and aggression. A future strategy of allocating individual RUSON to specific wards or departments to facilitate the development of professional relationships may confer similar mutual benefits of increased feelings of support for RUSON. This may also help with managing workload pressure and allowing registered staff more time to work at the higher end of their scope of practice, consistent with previous study findings (Sweet et al. 2023).

In our study, registered nurses and midwives valued the positive influence of the RUSON and RUSOM workforce on their ability to provide person‐centred care. Midwives also reported the positive influence of the RUSOM workforce on their sense of well‐being and mental health at work, and their intention to remain in the profession. This finding is important in light of the predicted nursing and midwifery workforce deficits and serious concerns about capacity to meet future workforce demand (Australian Institute of Health and Welfare 2024; Homer et al. 2024). Implementation of a RUSON and RUSOM model has the potential to positively impact retention of nurses and midwives to promote workforce sustainability (Willetts et al. 2021).

Our findings of improved perceptions of RUSON/M work readiness upon graduation are consistent with previous studies of undergraduate employment (Algoso et al. 2018, 2019; Gamroth et al. 2006; McLachlan et al. 2011) and strengthen the body of evidence in support of the RUSON/M role. However, the issue of *‘Placement poverty’* (O'Kane 2024, 6), caused by mandatory unpaid clinical placements, is known to deter students from completing undergraduate courses in professions such as nursing and teaching. A key recommendation from a review of the Australian higher education system completed by the Australian Universities Accord in 2024 is for the Australian Government ‘to work with tertiary education providers, state and territory governments, industry, business and unions to introduce financial support for unpaid work placements’(O'Kane 2024, 23). In response, the Australian Government has recently introduced the Commonwealth Prac Payment (CPP) to provide eligible domestic nursing, midwifery, teaching and social work students with some financial support while they're undertaking mandatory placements (Australian Government Department of Education 2025). There is an opportunity for future collaboration between universities, employers and government to devise and evaluate innovative models for employment or financial support of undergraduate healthcare students, particularly to address the workforce recruitment needs of rural, regional and underserved communities and to improve workplace readiness of graduates.

A limitation of this study was that it was conducted in only one of the many Victorian public health services that employ RUSON/M. However, the sampling strategy afforded by the multi‐method study design allowed deep exploration of the perspectives of both RUSON and RUSOM enrolled in undergraduate nursing and/or midwifery courses at different universities. In addition, the study sample included a diverse range of nurses and midwives in terms of clinical experience and expertise, seniority and role type, across a range of clinical contexts and differing styles of deployment within the workforce. A set of key recommendations for policy, practice and future research informed by this study are presented in Table 5.

**TABLE 5:** Policy, practice and research recommendations for future use of the Registered Undergraduate Students of Nursing or Midwifery employment model.

Area	Recommendation
Policy	Ensure effective clinical leadership and support relevant to the needs of employed Registered Undergraduate Students of Nursing or Midwifery within the workplace
	Map availability of clinical support resources for Registered Undergraduate Students of Nursing or Midwifery employed within health services
	Ensure employed Registered Undergraduate Students of Nursing or Midwifery are familiar with their direct line managers, clinical educators and clinical shift leaders in the workplace
	Ensure employed Registered Undergraduate Students of Nursing or Midwifery have clear pathways to access timely clinical support and debriefing
	Consider options to embed the employed Registered Undergraduate Students of Nursing or Midwifery role into the strategic health workforce plan
	Health service, university and government partners work together to secure long‐term funding for the Registered Undergraduate Students of Nursing or Midwifery role
Practice	Strengthen professional mentorship, delegation and supervision skills of nurses and midwives working with Registered Undergraduate Students of Nursing or Midwifery
	Support the professional development needs of registered nurses and midwives for mentorship, delegation and supervision of Registered Undergraduate Students of Nursing or Midwifery in clinical practice
	Clarify the professional scope of practice of Registered Undergraduate Students of Nursing or Midwifery in differing clinical contexts
	Support opportunities for Registered Undergraduate Students of Nursing or Midwifery employee development
	Identify opportunities for development of key skills that can be achieved by Registered Undergraduate Students of Nursing or Midwifery within their defined scope of practice in different clinical contexts
	Foster professional relationships between undergraduate employees and registered staff in the workplace
	Introduce and welcome employed undergraduate students to the clinical team each shift
	Consider allocation of employed undergraduate students to a specific clinical team for a designated period of time to promote integration into the team and acquisition of skills
Research	Conduct further research to inform, evaluate and further develop Registered Undergraduate Students of Nursing or Midwifery employment models
	Explore the perspectives of consumers on the impact of the Registered Undergraduate Students of Nursing or Midwifery employment models upon their experiences of care
	Collaborate with key partners to devise, pilot and evaluate innovative models for employment or financial support of undergraduate healthcare students in other professions

## 6 Conclusion

This study explored the support, information and resource needs of RUSON/M employed as a supplementary workforce within a health service using a combination of quantitative and qualitative research methods. The inclusion of participants from both nursing and midwifery disciplines has provided unique insights into critical differences in RUSON and RUSOM deployment within clinical teams that impacted the experiences of individual RUSON/M and the potential benefits of the RUSON/M role. As a result, we have a broader understanding of the potential for RUSON/M to be employed as a supplementary workforce within the Victorian healthcare system, how RUSON/M can be supported by both their employers and universities to successfully assimilate into the workforce and how utilisation of RUSON/M may improve retention of nurses and midwives to promote future workforce sustainability. Key findings from this research provide a deeper understanding of the importance of consistent workplace deployment of RUSON/M and integration of RUSON/M within existing nursing and midwifery teams, conducive to the development of mutually beneficial professional relationships.

## Author Contributions

V.J.W., T.B., A.E., S.P., D.K., N.M.P., L.S. and A.M.H. made substantial contributions to conception and design, or acquisition of data, or analysis and interpretation of data. V.J.W., T.B., S.P., D.K., N.M.P., L.S. and A.M.H. involved in drafting the manuscript or revising it critically for important intellectual content. V.J.W., T.B., S.P., D.K., N.M.P., L.S. and A.M.H. given final approval of the version to be published. Each author should have participated sufficiently in the work to take public responsibility for appropriate portions of the content. V.J.W., T.B., S.P., D.K., N.M.P., L.S. and A.M.H. agreed to be accountable for all aspects of the work in ensuring that questions related to the accuracy or integrity of any part of the work are appropriately investigated and resolved.

## Funding

This research was funded by a research grant awarded by The Victorian Nurses and Midwives Trust www.vnmt.org.

## Disclosure

Statistics: The statistics were checked prior to submission by an expert statistician, Dr. Tanita Botha tanita.botha@deakin.edu.au.

## Ethics Statement

This project was considered by the health service Human Research Ethics Committee and, having fulfilled the requirements of the National Statement on Ethical Conduct in Human Research (2007), was approved on 27 July 2023, reference number HREC/97017/VICBH‐2023‐379037. This approval was noted by the Deakin University Human Research Ethics committee on 11 September 2023, reference number 2023‐272.

## Conflicts of Interest

The authors declare no conflicts of interest.

## Data Availability

The data that support the findings of this study are available on request from the corresponding author. The data are not publicly available due to privacy or ethical restrictions.

## Appendix A Interview Guide

### RN/RM Interview Guide

Questions:

1. How long have you been working with RUSON/M at the health service?

*Prompt*:

*Have you experienced working with RUSON/M outside of …?*

2. How would you describe your experiences of working with RUSON/M?

*Prompt:*

*Have your experiences been generally positive or negative?*

*Why?*

3. Did you think that the skills learned by RUSON/M the first year of undergraduate training adequately prepare them for the demands of clinical work as a RUSON/M?

*Prompt:*

*What type of tasks are they most helpful with? What skills could be improved upon? Are they an asset towards achieving the daily workload on shift? Do they take a lot of time for supervision and support?*

4. How confident did you feel about working with RUSON/M when they first joined the workforce??

*Prompt:*

*For example, were you clear about their scope of practice? Did you feel comfortable to delegate and supervise RUSON/M in your role as a Registered Nurse/Registered Midwife?*

*What processes were put in place by health service to support you in your role as RN/RM to work with RUSON/M? Did they help you integrate the RUSON/M role into the team?*

5. Have you any concerns about working with RUSON/M?

*Prompt:*

*Do you feel that they receive adequate supervision and/or support from the nursing or midwifery education team, the ward or department managers or the registered nurses or midwives on shift?*

*What could be improved?*

6. Do you feel that there have been benefits of the RUSON/M role for you in your daily work as a Registered Nurse/Registered Midwife?

*Prompt:*

*What do you feel are the benefits of the RUSON/M role for the provision of patient care?*

*Have you experienced any negative factors from working with a RUSON/M?*

* Do you feel as though the RUSON/M are valued members of the team?*

*Would you recommend other undergraduate student nurses and/or midwives to work as a RUSON/M?*

7. Have you got any further insights/thoughts/comments or questions that you would like to share with us?

### RUSON/M Interview Guide

Questions:

1. How long have you been working as a RUSON or RUSOM?

*Prompt:*

*How far through are you with your undergraduate degree studies?*

*Have you experience of health or personal care work outside of?*

2. How would you describe your initial experiences of working as a RUSON or RUSOM at?

*Prompt:*

*Was the experience positive or negative?*

*What emotions did you experience when you first started as a RUSON or RUSOM? Was it nerve‐wracking, exhilarating, boring, enjoyable, etc.?*

3. Did your undergraduate training help you feel prepared for the demands of clinical work?

*Prompt:*

*How confident did you feel when you first started?*

*What parts of your undergraduate training at the University were helpful to support preparation for undergraduate students for RUSON or RUSOM work?*

*What could be improved by the University to support preparation for undergraduate students for RUSON or RUSOM work?*

4. How helpful were the induction, orientation and/or training processes facilitated by towards your practice as a RUSON or RUSOM?

*Prompt:*

*What type of orientation sessions did you attend when you first started working as a RUSON or RUSOM?*

*Did they help you settle into your RUSON or RUSOM role?*

5. What type of support, supervision or mentorship do you receive to help you in your role as a RUSON or RUSOM?

*Prompt:*

*Do you receive support from the nursing or midwifery education team/the ward or department managers/the registered nurses or midwives on shift/other employees, such as medical, allied health, ancillary or clerical staff?*

*What type of support, supervision or mentorship do you find most helpful?*

*What could be improved?*

6. Do you feel that there have been benefits of the RUSON or RUSOM role for you personally?

*Prompt:*

*What do you feel are the benefits of the RUSON or RUSOM role for undergraduate students?*

*Have you experienced any negative factors from working as a RUSON or RUSOM?*

*Do you feel like a valued member of the team when you work as a RUSON or RUSOM?*

*Would you recommend other undergraduate student nurses and/or midwives to work as a RUSON or RUSOM?*

7. Have you got any further insights/thoughts/comments or questions that you would like to share with us?
